# Aligned Polyhydroxyalkanoate Blend Electrospun Fibers as Intraluminal Guidance Scaffolds for Peripheral Nerve Repair

**DOI:** 10.1021/acsbiomaterials.2c00964

**Published:** 2023-02-27

**Authors:** Caroline S. Taylor, Mehri Behbehani, Adam Glen, Pooja Basnett, David A. Gregory, Barbara B. Lukasiewicz, Rinat Nigmatullin, Frederik Claeyssens, Ipsita Roy, John W. Haycock

**Affiliations:** † Department of Materials Science & and Engineering, The University of Sheffield, Sheffield S3 7HQ, United Kingdom; ‡ The Electrospinning Company, Unit 5, Zephyr Building, Eighth St., Harwell Campus, Harwell, Didcot OX11 0RL, United Kingdom; § School of Life Sciences, College of Liberal Arts and Sciences, University of Westminster, London W1B 2HW, United Kingdom

**Keywords:** electrospun fibers, nerve regeneration, peripheral nerves, polyhydroxyalkanoates, topographical guidance

## Abstract

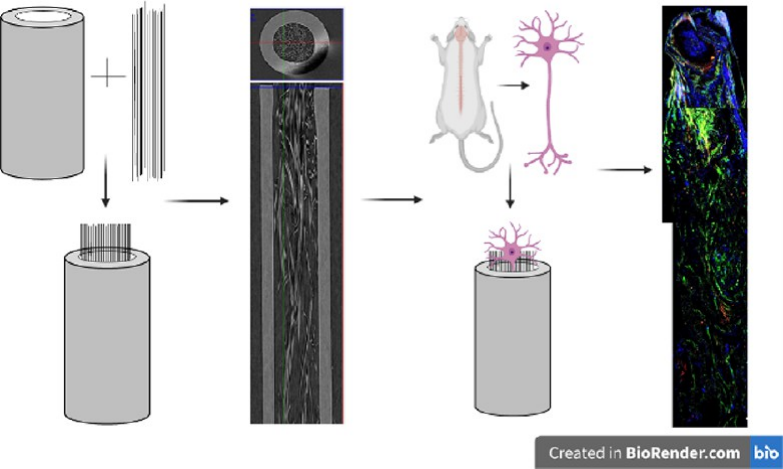

The use of nerve guidance conduits (NGCs) to treat peripheral nerve injuries is a favorable approach to the current “gold standard” of autografting. However, as simple hollow tubes, they lack specific topographical and mechanical guidance cues present in nerve grafts and therefore are not suitable for treating large gap injuries (30–50 mm). The incorporation of intraluminal guidance scaffolds, such as aligned fibers, has been shown to increase neuronal cell neurite outgrowth and Schwann cell migration distances. A novel blend of PHAs, P(3HO)/P(3HB) (50:50), was investigated for its potential as an intraluminal aligned fiber guidance scaffold. Aligned fibers of 5 and 8 μm diameter were manufactured by electrospinning and characterized using SEM. Fibers were investigated for their effect on neuronal cell differentiation, Schwann cell phenotype, and cell viability *in vitro*. Overall, P(3HO)/P(3HB) (50:50) fibers supported higher neuronal and Schwann cell adhesion compared to PCL fibers. The 5 μm PHA blend fibers also supported significantly higher DRG neurite outgrowth and Schwann cell migration distance using a 3D *ex vivo* nerve injury model.

## Introduction

Peripheral nerve injuries (PNIs), commonly arising from trauma incidents, cause long-term disability and affect patient quality of life.^1^ Peripheral nerves have regeneration capabilities, with a regeneration rate of up to 1 mm a day.^2^ However, axonal damage is often too severe for successful regeneration to occur, and surgical intervention is often required to restore nerve functionality and sensation.^3^ Autografts are the current “gold standard” for peripheral nerve repair (PNR) and are used to bridge large injury gaps (20–50 mm).^4^ However, issues with autograft use such as donor site morbidity and a lack of donor nerve have led to the increased use of nerve guidance conduits (NGCs).^1^ NGCs are entubulation devices with hollow tubular designs that have clinical success bridging gaps of 10–30 mm.^4^ Although a favorable alternative to autografting, current NGCs have limited success due to the type of biomaterial used, simplistic design, and lack of guidance cues/surface topography, which are present in nerve autografts.^5^ Therefore, several approaches such as novel biomaterials to manufacture NGCs, changing the conduit design by incorporating porosity and channels, and addition of intraluminal guidance scaffolds manufactured from electrospun fibers are currently being explored.^6^

The addition of aligned electrospun fibers to the NGC lumen is a novel approach for improving current nerve guidance devices.^7^ Fibers are used to replace and/or support the formation of the fibrin cable, the support structure for Schwann cell proliferation and migration to the distal stump following injury.^4^ Most recently, Frost *et al.* reported significant nerve regeneration when using a fiber-filled nerve guidance conduit compared to a hollow tube.^8^

When developing a fiber scaffold for nerve repair, one must consider three important factors: fiber alignment, fiber diameter, and choice of material for the scaffold. Previous studies have shown that aligned fiber scaffolds increase Schwann cell attachment, promote an elongated cell morphology, and induce a more mature Schwann cell phenotype compared to randomly orientated fiber scaffolds.^9^ By promoting a mature, myelinating Schwann cell phenotype, aligned fiber scaffolds could increase nerve regeneration rate *in vivo*.^10^ Aligned fiber scaffolds have also been shown to significantly increase neurite outgrowth and Schwann cell migration lengths from dorsal root ganglia (DRG)^11^ and promote successful regeneration of axons across a 17 mm nerve gap *in vivo*.^12^

Fiber diameter is another important consideration. Nanometer fiber scaffolds have been reported on using RT4-D6P2T Schwann cells,^13^ C17.2 stem cells,^14^ and dorsal root ganglia.^15^ More recently, it has been shown that aligned fibers in the micrometer range, compared to the nanometer range, are more effective at promoting neurite outgrowth from neuronal cells and DRGs *in vitro*.^16,17^ The biomaterial used to manufacture both nerve guidance conduits and intraluminal scaffolds is also important. Some natural materials have biological and mechanical properties similar to native nerve tissue. However, issues are evident with processing and batch-to-batch variability.^18^ Synthetic materials can be readily processed by a number of different manufacturing techniques, with mechanical and degradation properties tailored. To date, a limited range of polyester materials have been used that are brittle and degrade slowly into acidic products.^1^

The use of polyhydroxyalkanoates (PHAs) can eliminate concerns associated with current FDA-approved devices made from synthetic polyesters. PHAs are a family of naturally derived polyesters synthesized by bacterial fermentation.^19^ A wide range of PHAs can be manufactured that give a broad range of selectable biocompatible properties.^20^ PHAs are reported to support cell adhesion and growth, are biodegradable, have controllable surface erosion, have less acidity from degradation products, have tunable mechanical properties, and have already been researched thoroughly for nerve tissue engineering investigations. Previously, Prabhakaran *et al.* investigated poly(3-hydroxybutyrate-*co*-3-hydroxyvalerate) (PHBV) nanofibers for peripheral nerve repair, investigating PC12 neuronal cell biocompatibility and neurite extension lengths.^21^

Two PHAs of interest, poly(3-hydroxybutyrate) (P(3HB)) and poly(3-hydroxyoctanoate) (P(3HO)), have been investigated as NGC materials in PNR.^22,23^ However, it has been reported that P(3HB) nerve guidance conduits, although biocompatible, are often too brittle, whereas P(3HO) nerve guidance conduits, although elastomeric in nature, require surface treatment/modification to improve cellular adhesion and neuroregeneration for long-term implantation.^22,24^ Therefore, blending P(3HB) and P(3HO) together offers an opportunity for the development of a biocompatible blend with the desirable elastomeric properties. This approach has been investigated for a range of tissue engineering applications.^20,25^

We have previously fabricated and fully characterized blends of P(3HO)/P(3HB) for nerve tissue engineering applications in the form of solvent casted films. Films containing larger quantities of P(3HB) in the blend, such as P(3HO)/P(3HB) (25:75) and P(3HO)/P(3HB) (50:50), significantly supported NG108-15 neuronal cell viability and differentiation compared to the P(3HO)/P(3HB) (75:25) blend.^26^ The P(3HO)/P(3HB) (25:75) blend was further investigated for its potential as an intraluminal guidance scaffold and was fabricated into aligned micrometer fibers for peripheral nerve repair.^17^ Although promising, the P(3HO)/P(3HB) (25:75) blend has a significantly higher Young’s modulus than native nerve tissue (143.40 ± 2.16 MPa compared to 0.5 MPa), which could hinder optimum nerve regeneration *in vivo.*(26,27) Therefore, we have further investigated the P(3HO)/P(3HB) (50:50) blend for its potential as an intraluminal guidance scaffold because of its previously reported excellent biocompatibility, ability to significantly support NG108-15 neuronal cell viability and differentiation, and lower Young’s modulus of 21.83 ± 0.95 MPa.^26^ Our previous work investigating the P(3HO)/P(3HB) (25:75) blend did not investigate the effect of primary Schwann cell responses, such as metabolic activity, viability, and phenotype, nor did it investigate dorsal root ganglia (DRG) axon outgrowth or Schwann cell migration lengths in a 3D system.^16,26^ Therefore, we are the first to investigate PHA blend fibers for their potential as intraluminal guidance scaffolds using a novel 3D *ex vivo* nerve injury model using DRG explants.^7^

PHA fibers were manufactured by electrospinning, and diameters of 5 and 8 μm chosen, as these diameters have previously been shown to significantly promote NG108-15 neuronal cell neurite outgrowth, Schwann cell phenotype, cell viability, and DRG neurite outgrowth with concomitant Schwann cell migration compared to 1 μm PCL fibers, which were also investigated in the study.^4^ Polymer fibers were characterized by scanning electron microscopy for fiber diameter, alignment, and density. The effect of material and fiber diameter was investigated *in vitro* using NG108-15 neuronal cells and primary rat Schwann cells, investigating neuronal cell differentiation, Schwann cell phenotype, and cell viability. To assess the blend as a potential intraluminal guidance scaffold, fibers were inserted into 3D printed nerve guidance conduits and investigated using a novel 3D *ex vivo* testing model, which has been carried out for the first time with PHA fibers in this study.^7^

## Materials and Methods

### Production and Characterization of PHAs

P(3HB) and P(3HO) were produced via bacterial fermentation. P(3HB) was produced by *Bacillus cereus* SPV using glucose as the sole carbon source as per conditions described.^28^ P(3HO) was produced by *Pseudomonas mendocina* CH50 using sodium octanoate as the carbon source as detailed previously.^29^

### Fabrication, Characterization, and Preparation of Aligned Electrospun Fibers and Films

Aligned PHA blend and PCL fibers were fabricated by electrospinning and characterized to confirm fiber diameter, alignment, and fiber frequency.^16^ Electrospinning was performed using a setup containing a grounded, 6 cm diameter, rotating cylindrical collector (IKA, UK); a high-voltage power supply (Genvolt, UK); and a 1 mL syringe with a 20G blunt needle, containing the polymer solution, connected to a syringe pump (WPI, USA). Fibers were collected on aluminum foil and analyzed for fiber diameter, frequency, and alignment using an FEI Sirion field emission gun scanning electron microscope and an electron beam with energy of 20 kV.^16^ PCL fibers of 5 and 8 μm were fabricated as per the conditions previously described.^16^ For cell culture, fiber mats were cut into 20 × 20 mm squares, held in place with medical-grade 316 stainless steel rings (inner diameter = 13 mm, outer diameter = 24 mm), and sterilized with 70% ethanol in PBS prior to cell seeding.^16^ Films of both the PHA blend and PCL were prepared by spin coating for water contact analysis and AFM analysis. One hundred fifty microliters of 10 wt % polymer was dropped onto a 13 mm^2^ glass coverslip. Samples were spun for 3000 rpm for 30 s at room temperature using a spin coater. Samples were left to dry, and films were peeled off coverslips for analysis.

### Water Contact Angle

Water contact angle was measured with a Krüss GmbH drop shape analyzer (DSA100) by placing 5 μL of distilled water on the test surface. The contact angle of surfaces was measured and calculated using the DSA100 software.

### Atomic Force Microscopy (AFM)

AFM was performed in tapping mode with SCANASYST-AIR probes under ambient conditions on a Bruker Dimension Icon AFM. Films of PCL or PHA blends were cast onto cover glasses and then stuck to a magnetic AFM support. Different areas of the samples were then analyzed and characterized in the Bruckers NanoScope Analysis software (Version 2.0). Data were further averaged by weight dependent on the surface area measured to obtain accurate Rq (root mean squared roughness), Ra (roughness average), and adhesion values (how adhesive the polymer film is to the tip of the AFM cantilever).

### Protein Adsorption Assay

A protein adsorption assay was performed on fiber scaffolds to determine the amount of adsorbed proteins from undiluted fetal bovine serum (FBS) as previously described.^20^ Briefly, fiber scaffolds were cut into 10 × 10 mm squares and incubated with 400 μL of undiluted fetal bovine serum (FBS) at 37 °C for 24 h. Samples were washed once with PBS, removed, and placed into a new well plate. 1 mL of 2% sodium dodecyl sulfate (SDS) in PBS was added to each scaffold for 24 h at room temperature under shaking conditions. A bicinchoninic acid assay was then performed to measure absorbance at 562 nn.^20^

### NG108-15 Neuronal Cell Culture

NG108-15 (ECACC 88112303) neuronal cells were cultured in Dulbecco’s modified Eagle’s medium (DMEM) supplemented with 10% fetal calf serum, 1% penicillin/streptomycin, 1% glutamine, and 0.5% amphotericin B at 37 °C and 5% CO_2_ and used for experiments as passage 15. A total of 30,000 NG108-15 neuronal cells were seeded onto fiber scaffolds for 6 days, with the medium replaced with serum-free Dulbecco’s modified Eagle’s medium (DMEM) after 2 days to induce neurite outgrowth from neuronal cells.^16^

### Isolation and Culture of Primary Schwann Cells

Primary Schwann cells were isolated from adult male Wistar rat sciatic nerves and cultured in specialized DMEM containing d-valine to inhibit fibroblast growth up to passage 2 to confirm Schwann cell purity.^30^ A total of 60,000 Schwann cells were seeded onto polymer samples in DMEM containing 10% fetal calf serum, 0.01% forskolin (Sigma Aldrich), 1% penicillin/streptomycin, 1% glutamine, and 0.5% amphotericin B at 37 °C with 5% CO_2_ for 6 days, and the medium was replaced after 3 days in culture.^30^

### Metabolic Activity of NG108-15 Neuronal Cells and Primary Schwann Cells

The metabolic activity of NG108-15 neuronal cells and primary Schwann cells on scaffolds was measured using an MTT (3,4,5-dimethylthiazol-2,5-diphenyl tetrazolium bromide) assay, as previously described.^31^ Briefly, cells were seeded onto substrates, and at each time point (24, 72, and 144 h), the culture medium was removed and replaced with 1 mL of MTT (0.5 mg/mL) solution. Samples were incubated for 1 h at room temperature, the MTT solution was removed, and 600 μL of acidified isopropanol (5 μL of HCl in 5 mL of isopropanol) was added.^31^ A BIO-TEK ELx 800 microplate reader was used to measure absorbance at 540 nm and referenced at 630 nm.^31^

### Live/Dead Analysis of NG108-15 Neuronal Cells and Primary Schwann Cells

The number of live and dead cells per sample and the cell viability were confirmed by a live/dead assay. A serum-free culture medium containing 0.001% SYTO 9 (Invitrogen) and 0.0015% propidium iodide (Invitrogen) was added to samples and incubated at 37 °C and 5% CO_2_ for 30 min.^16^ An upright Zeiss LSM 510 confocal microscope was used to visualize samples, with three fields of view taken per sample and analyzed to calculate cell numbers (live versus dead cells) and cell viability (live/dead cell ratio as percentage).^16^ Cells were counted using an ITCN cell counter plugin on the ImageJ NIH software.^32^

### *Ex Vivo* 3D Cell Culture

The ability of the fibers to support Schwann cell migration and neurite outgrowth distance from DRGs was investigated using a 3D *ex vivo* fiber testing model.^7^ Briefly, nerve guidance conduits were manufactured from polyethylene glycol (PEG)—5 mm length, 1.1 mm diameter, and 250 μm wall thickness—using microstereolithography as previously described.^31^ Six to seven thousand fibers were threaded into conduit lumens using a sewing needle (20G). Conduits containing fibers were placed into bespoke holders and placed in a metal grid in a six-well cell culture plate. Dorsal root ganglia were isolated from male Wistar rat spinal columns, and nerve roots were trimmed off and placed on top fibers inside NGCs to attach for 15 min at 37 °C.^7^ Once attached, DRGs were submerged with Dulbecco’s modified Eagle’s medium (DMEM) supplemented with 10% fetal calf serum, 1% penicillin/streptomycin, 1% glutamine, and 0.5% amphotericin B and cultured at 37 °C and 5% CO_2_ for 21 days.^7^

### Mechanical Testing of Fibers

Dry electrospun fiber samples, approximately 6000–7000 fibers, were cut into 10 × 8 mm square samples for testing in bundles as previously described.^7^ Fiber bundles were positioned vertically and were clamped to a tensiometer (Bose ElectroForce test instruments, Minnesota, USA) using a 405 N load cell. The weight, density, and thickness of the fiber bundles were recorded before testing to calculate the area and cross-sectional area. An extension rate of 0.2 mm/s and a maximum extension of 8 mm were used. The ultimate tensile strength (UTS) and Young’s modulus (YM) were calculated for each fiber type and diameter.

### Microcomputed Tomography (MicroCT) of Fibers in NGCs

MicroCT was performed on a Skyscan 1272 (Bruker) as previously described.^33^ The percentage volume occupancy of fibers threaded into 3D printed nerve guide conduits was determined by the following equation:
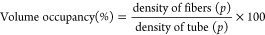
1

The mass of fiber bundles and tubes was taken prior to threading, and the volume of fibers was calculated by quantifying microCT micrographs using ImageJ.^34^

### Immunolabeling of NG108-15 Neuronal Cells, Schwann Cells, and Dorsal Root Ganglia

To visualize cell specific proteins, cells and DRG explants were immunolabeled by fixing samples in 4% (v/v) paraformaldehyde (for 20 min) and permeabilized with 0.1% Triton X-100 (for 30 min). 3% bovine serum albumin (BSA) (for 30 min) was then added to block unreactive binding sites.^16^ NG108-15 neuronal cells, and neurites outgrown from DRG explants, were labeled for βIII-tubulin (neurite marker) using a mouse anti-β III-tubulin antibody (1:500 dilution in 1% BSA) (Promega, UK) and incubated for 24 h at 4 ° C. After a wash in PBS, a Texas Red-conjugated anti-mouse IgG antibody was added to samples (1:100 dilution in 1% BSA) (Vector Labs, USA) for 2 h at room temperature.^16^ Primary Schwann cells in monoculture and co-culture from DRG explants were labeled for S100β using a polyclonal rabbit anti-S100β antibody (1:250 in 1% BSA) (Dako, Denmark) and incubated at 4 °C for 24 h followed by a FITC-conjugated secondary anti-rabbit IgG antibody (1:100 dilution in 1% BSA) (Vector Labs, USA) for 2 h at room temperature. Nuclei of all cells were stained with 4′,6-diamidino-2-phenylindole dihydrochloride (DAPI) (Sigma Aldrich) (1:500 dilution in PBS) for 30 min at room temperature prior to imaging samples using an upright Zeiss 510 confocal microscope.^16^ In the *ex vivo* model, fibers were carefully removed from PEG tubes and placed into cell culture well plates before staining, washing, and imaging using an upright Zeiss LSM 510 confocal microscope.^7^

### Neurite Outgrowth and Primary Schwann Cell Morphology Assessment

Three different parameters were analyzed for neurite outgrowth: percentage of neurite bearing cells, number of neurites per neuron, and the average neurite length.^16^ Images were analyzed using ImageJ (NIH), and neurites were traced using the Neuron J plugin tracer software from the cell body to the neurite tip.^35^ The average Schwann cell length and aspect ratio^36^ were calculated as previously described using the ruler tool on NIH ImageJ.^34^

### Statistical Analysis

GraphPad Instat (GraphPad Software, USA) was used to perform statistical analysis. One-way analysis of variance (ANOVA; *p* < 0.05) was conducted to analyze the differences between data sets incorporating Tukey’s multiple-comparisons test if *p* < 0.05. Two-way analysis of variance (*p* < 0.05) was conducted to analyze the differences between data sets when assessing live/dead cell numbers incorporating Sidak’s multiple-comparisons test if *p* < 0.05. Data were reported as mean ± SD, *p* < 0.05. Each experiment was performed three independent times with each sample repeated three times as *n* = 3.

## Results

### Water Contact Angle of P(3HO)/P(3HB) and PCL Films

Water contact analysis showed that the PHA blend films had a significantly lower contact angle of 84.3 ± 1.6° compared to the PCL films, which exhibited a water contact angle of 104.9 ± 2.1°. This indicates that the PHA blend films exhibit a more hydrophilic nature, whereas PCL is more hydrophobic.

### Adhesion and Surface Roughness Properties of P(3HO)/P(3HB) and PCL Films Using Atomic Force Microscopy

**Figure 1:**
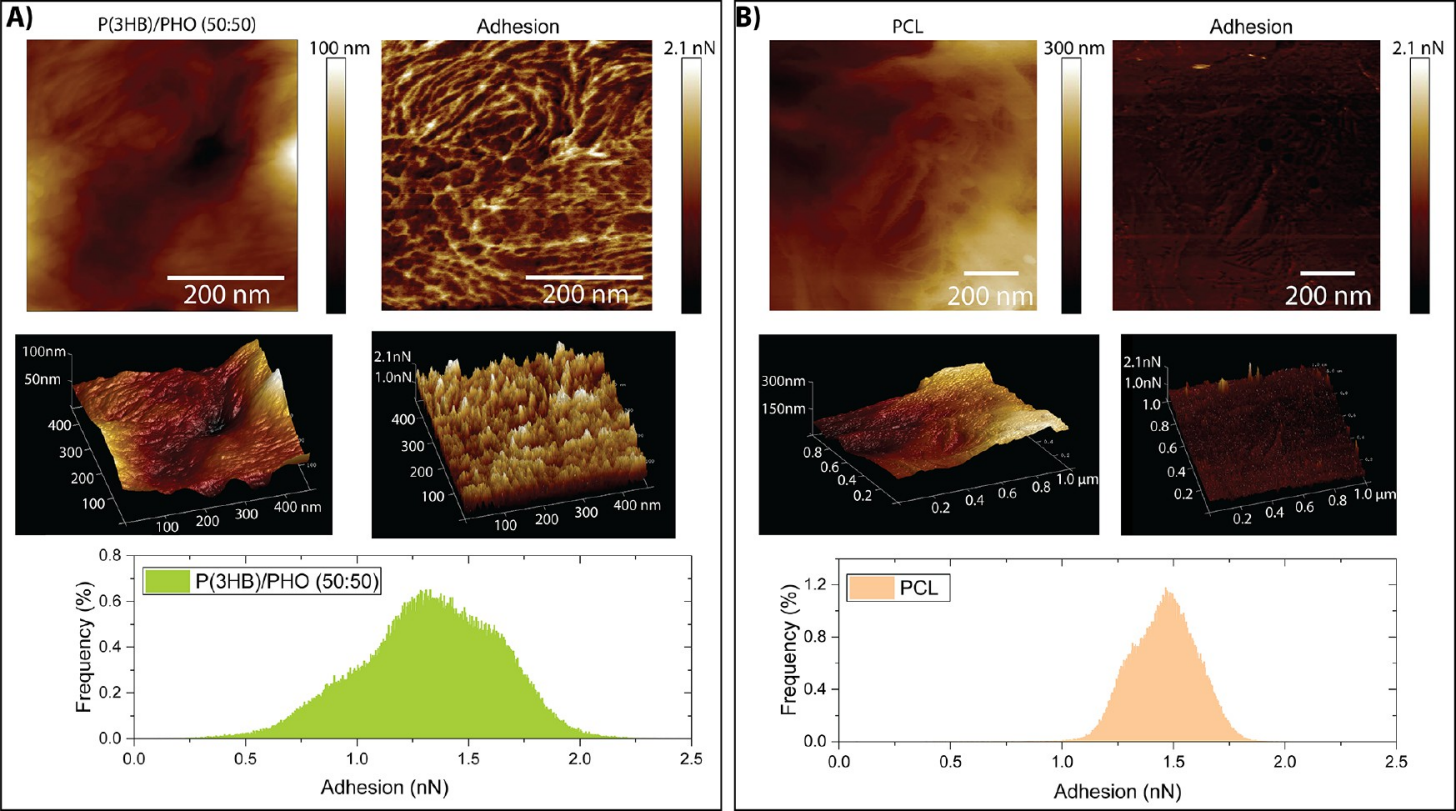
Representative AFM surface topography micrographs, AFM adhesion maps, and 3D renderings and representative histograms showing the adhesion distribution of (A) P(3HB)/P(3HO) (50:50) and (B) PCL films.

Representative AFM surface topography micrographs and 3D rendered representations of P(3HO)/P(3HB) (50:50) and pure PCL films revealed differences in topography and surface roughness between the two materials. Micrographs were quantified to calculate the typical root mean square (Rq) and roughness average (Ra) values to determine the surface roughness of the films. PHA blends exhibited a significantly higher Rq value, 154.59 ± 62.61 nm, and Ra value, 125.93 ± 55.81 nm, compared to PCL films, which had an average Rq value of 12.24 ± 2.92 nm and Ra value of 9.35 ± 2.48 nm (Table 1). Areas of low and high adhesion are seen in representative AFM adhesion maps (Figure 1A,B) of the PHA blend, representative of the P(3HB) and the P(3HO), whereas adhesion was consistent across the whole PCL surface. Adhesion maps were quantified, confirming a significantly higher adhesion number for PHA blends, 4.38 ± 0.54 nN, compared to PCL films, 1.18 ± 0.18 nN, of a Bruker Silicon Nitride ScanAsyst AFM tip to the sample (Table 2). Representative histograms (Figure 1A,B) showing the adhesion distribution across the adhesion maps indicated a larger range of adhesion values in the PHA blend compared to PCL films due to the immiscibility of P(3HO) and P(3HB) in the polymer solution.

**Table 1:** Values of Materials Quantified from AFM Surface Topography Micrographs

material	Rq (nm)	Ra (nm)	adhesion (nN)	overall surface area analyzed (μm^2^)
P(3HB)/P(3HO) (50:50)	154.59 ± 62.61	125.93 ± 55.81	4.38 ± 0.54	8.46
PCL	12.24 ± 2.92	9.35 ± 2.48	1.18 ± 0.18	2.75

**Table 2:** Summary of Electrospinning Conditions Used to Manufacture Aligned Fibers

material	concentration (w/v %)	flow rate (mL/h)	voltage (kV)	distance (cm)	speed of collector (rpm)	diameter of fibers (μm ± SD)
P(3HO)/P(3HB) (50:50)	8	3	12.5	15	2000	4.97 ± 0.39
P(3HO)/P(3HB) (50:50)	8	3	12.5	10	2000	7.86 ± 0.49
PCL	10	4	15	20	2000	5.07 ± 0.96
PCL	20	6	18	20	2000	8.08 ± 0.43

### Protein Adsorption on P(3HO)/P(3HB) and PCL

**Figure 2:**
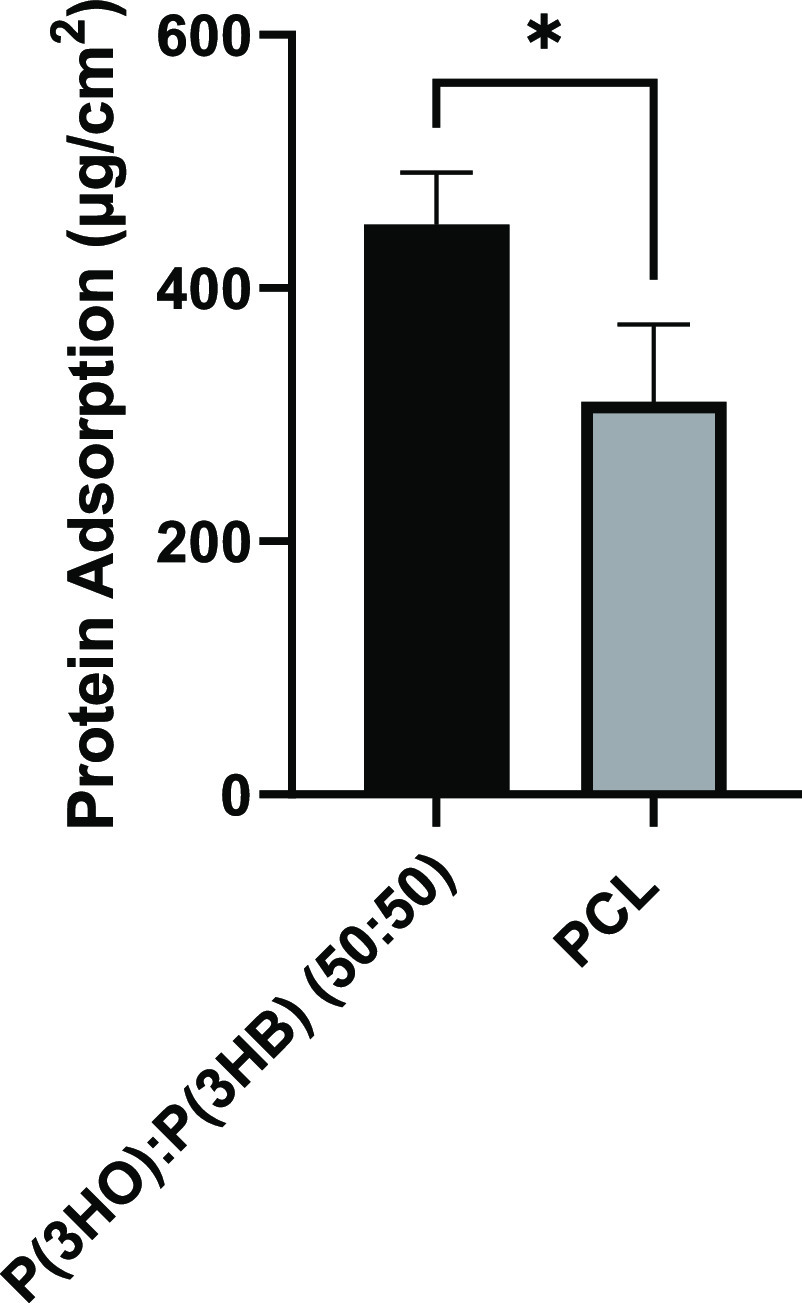
Protein adsorption of the P(3HO)/P(3HB) (50:50) blend and PCL films (mean ± SD, *n* = 3 independent experiments; *p < 0.05).

The total amount of protein adsorbed onto P(3HO)/P(3HB) and PCL was determined using a protein adsorption assay (Figure 2). P(3HO)/P(3HB) (50:50) had a significantly higher amount of protein adsorption (450.05 ± 41.23 μg/cm^2^) compared to the PCL (310.76 ± 61.34 μg/cm^2^). This indicates that changes in surface roughness and increased hydrophilicity from the PHA blend can increase protein adsorption.

### Characterization of Aligned Fiber Scaffolds

**Figure 3:**
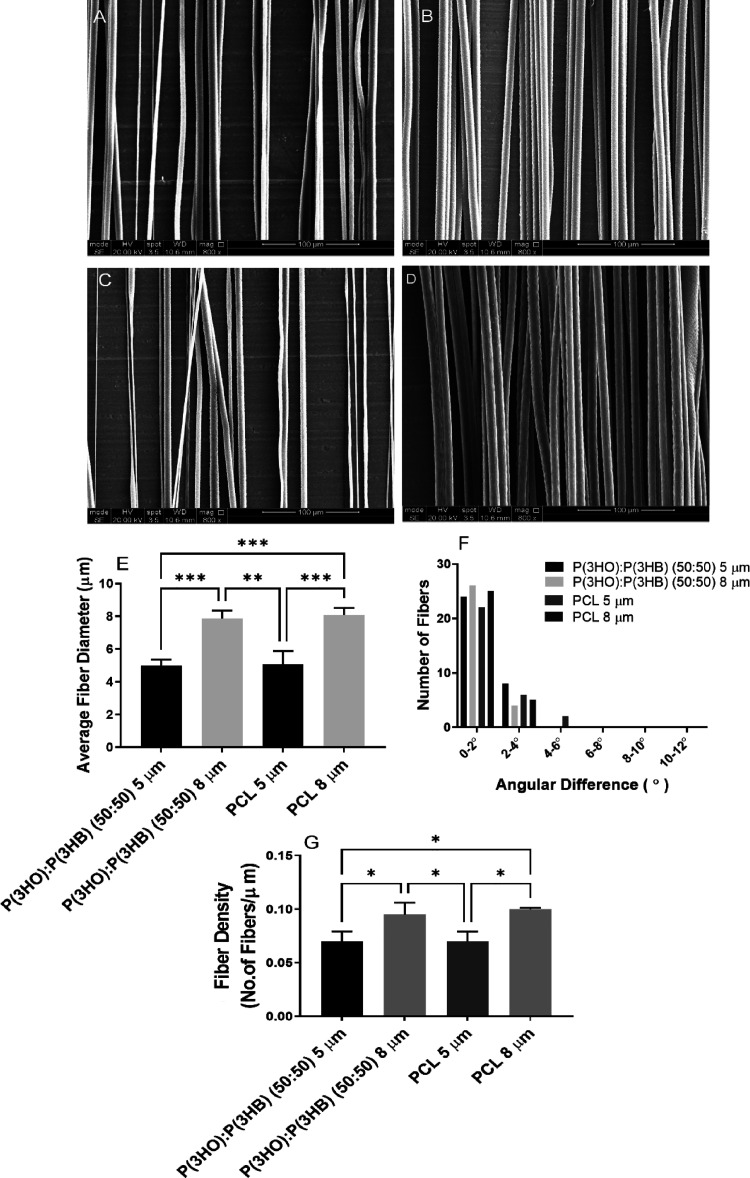
Scanning electron micrographs of (A) P(3HO)/P(3HB) (50:50) 5 μm fibers, (B) P(3HO)/P(3HB) (50:50) 8 μm fibers, (C) PCL 5 μm fibers, and (D) PCL 8 μm fibers (scale bar =100 μm). (E) Average diameter of polymer fiber data (mean ± SD, *n* = 3 independent experiments). An average of 30 fibers was assessed for each fiber condition. (F) Histogram to show the angular variance of fibers and (G) fiber density of electrospun mats (mean ± SD, *n* = 3 independent experiments; *p < 0.05 and ***p < 0.001). The number of fibers per micrometer was determined by counting the number of fibers per 100 μm.

Aligned PCL and PHA blend fibers were fabricated by electrospinning; conditions are summarized in Table 2. Fibers were imaged using SEM, and micrographs (Figure 3A–D) were quantified to determine fiber diameter, alignment, and density. Figure 3E confirms the PHA blend fiber diameters as 4.97 ± 0.38 and 7.86 ± 0.49 μm. Fiber alignment was confirmed by determining the angular variance between fibers and is presented in a histogram (Figure 3F). For all groups, 73% and above of all fibers were in the 0–2° group, with very few fibers present in the 2–4 and 4–6° groups, and no fibers were detected in the higher degree groups. The 8 μm fibers were found to have a significantly higher fiber frequency compared to the 5 μm fibers. Fiber frequencies for P(3HO)/P(3HB) (50:50) 5 and 8 μm fibers and PCL 5 and 8 μm were 0.07 ± 0.01, 0.09 ± 0.01, 0.07 ± 0.01, and 0.10 ± 0.01 fibers/μm, respectively.

### NG108-15 Neuronal Cell Metabolic Activity on Aligned Fibers with Varying Diameters

**Figure 4:**
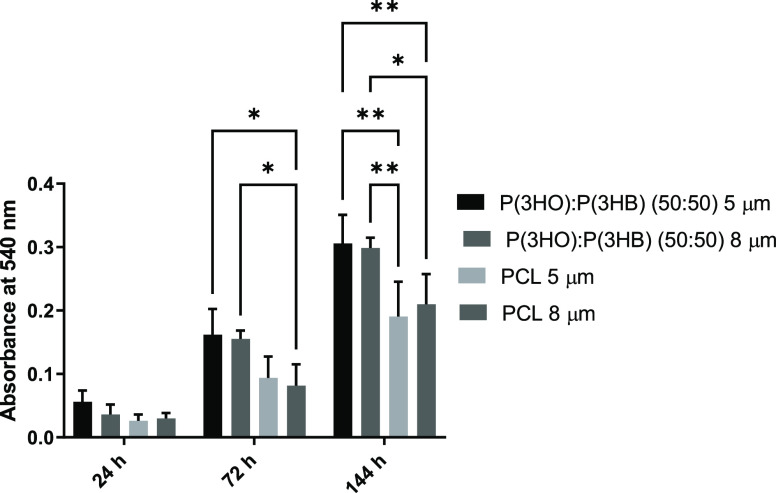
Metabolic viability assay (MTT) of NG108-15 cells on 5 and 8 μm P(3HO)/P(3HB) (50:50) and PCL fibers at 24, 72, and 144 h of culture (mean ± SD, *n* = 3 independent experiments; *p < 0.05 and **p < 0.01).

The metabolic activity of NG108-15 neuronal cells cultured on fibers was determined via an MTT assay (Figure 4). No significant difference was detected on samples after 24 h in culture. However, after 72 h, PHA blend fibers supported significantly higher NG108-15 neuronal cell metabolic activity compared to the PCL 8 μm fibers. After 144 h in the culture medium, both the 5 and 8 μm PHA blend fibers supported significantly higher NG108-15 neuronal cell metabolic activity compared to both the 5 and 8 μm PCL fibers.

### NG108-15 Neuronal Cell Viability on Aligned Fibers with Varying Diameters

**Figure 5:**
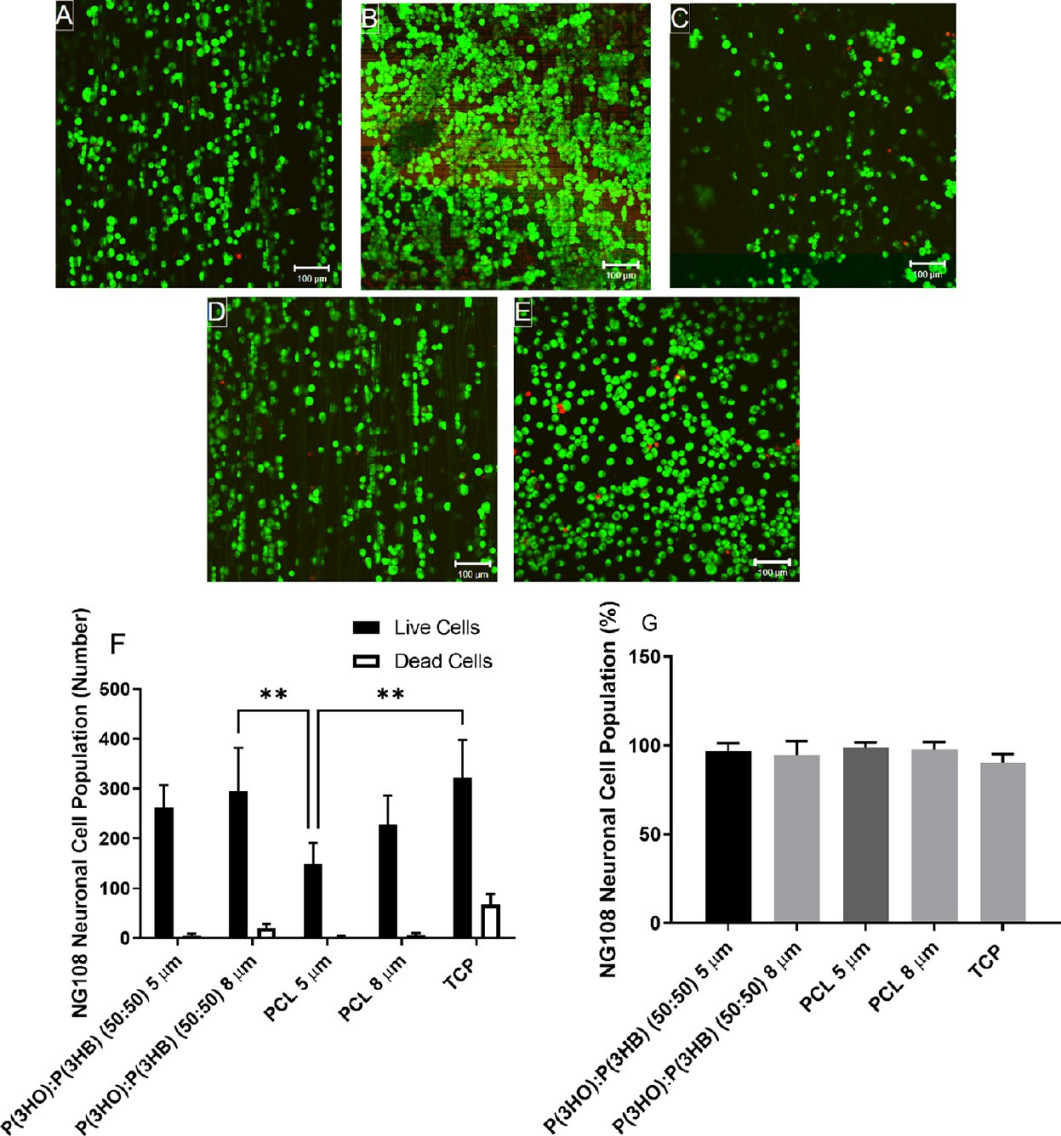
Confocal micrographs illustrating NG108-15 neuronal cell viability cultured on (A) P(3HO)/P(3HB) (50:50) 5 μm fibers, (B) P(3HO)/P(3HB) (50:50) 8 μm fibers, (C) PCL 5 μm fibers, (D) PCL 8 μm fibers, and (E) tissue culture plastic (scale bar = 100 μm). Cells labeled with SYTO 9 (green = live cells) and propidium iodide (red = dead cells). (F) Number of live cells versus dead cells per sample and (G) live cell analysis expressed as a percentage (mean ± SD, *n* = 3 independent experiments; *p < 0.05 and **p < 0.01).

The effect of material chemistry and fiber diameter on NG108-15 neuronal cell viability was investigated. Confocal micrographs identified that neuronal cells attached and aligned on the fibers (Figure 5A–D), whereas cells adhered and proliferated in a random orientation on tissue culture plastic (TCP) (Figure 5E). Very few dead cells were observed on samples. Figure 5F shows that significantly higher numbers of live cells were observed on the P(3HO)/P(3HB) (50:50) 8 μm fibers (295 ± 87 cells) and TCP control (323 ± 76 cells) as compared to the PCL 5 μm fibers (149 ± 42 cells). The number of live cells observed on the PCL 8 μm fibers and P(3HO)/P(3HB) (50:50) 5 μm fibers was 228 ± 58 and 262 ± 46 cells, respectively. With regard to cell viability (Figure 5G), cell viabilities of >95% were detected on all fiber conditions, and a cell viability of 90.38 ± 4.82% was detected on the TCP control as a result of the increased numbers of dead cells. No significant differences were identified between experimental groups.

### NG108-15 Neuronal Cell Differentiation on Aligned Fibers with Varying Diameters

**Figure 6:**
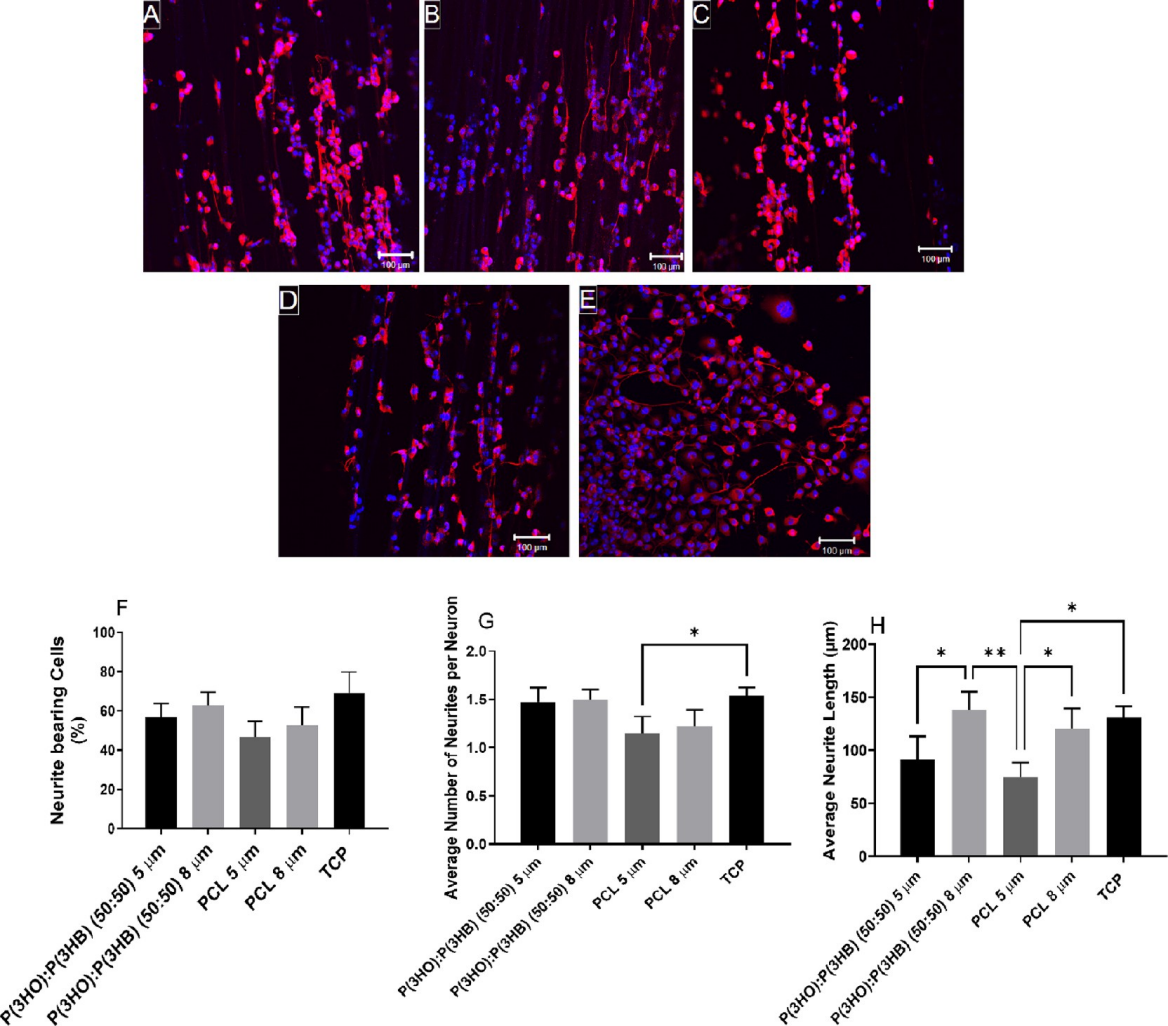
Confocal micrographs of NG108-15 neuronal cells immunolabeled for βIII-tubulin and DAPI on (A) P(3HO)/P(3HB) (50:50) 5 μm fibers, (B) P(3HO)/P(3HB) (50:50) 8 μm fibers, (C) PCL 5 μm fibers, (D) PCL 8 μm fibers, and (E) tissue culture plastic control (scale bar = 100 μm). (F) The percentage of neurite bearing neuronal cells after 6 days in culture. (G) The average number of neurites expressed per neuron. (H) Average neurite length per condition after 6 days in culture (mean ± SD, *n* = 3 independent experiments; *p < 0.05 and **p < 0.01).

NG108-15 neuronal cells cultured on aligned fiber scaffolds were labeled for βIII-tubulin and DAPI (to identify nuclei) to quantify neurite formation. Neuronal cells adhered and aligned to the fiber scaffolds (Figure 6A–D). Outgrowing neurites aligned to the fiber scaffolds could also be visualized, whereas neurite outgrowth was in a random orientation on TCP. Longer neurites could be visualized on the PHA blend fibers compared to the PCL fibers. The percentage of neuronal cells bearing neurites (Figure 6F) for P(3HO)/P(3HB) (50:50) 5 and 8 μm fibers, PCL 5 and 8 μm fibers, and the TCP control was 56.88 ± 6.91, 62.86 ± 6.79, 46.89 ± 7.80, 52.66 ± 9.37, and 69.29 ± 10.62%, respectively. No significant differences were detected between data sets. The TCP control had a significantly higher average number of neurites per neuron from neuronal cell bodies (1.54 ± 0.08, Figure 6G) compared to 5 μm PCL fibers (1.15 ± 0.17). However, no significant differences were detected between the PHA and PCL materials and different fiber diameters.

The average neurite length (Figure 6H) was determined for each material and fiber diameter. Average neurite lengths on P(3HO)/P(3HB) (50:50) 8 μm fibers (137.94 ± 17.35 μm) were significantly longer than those observed on the P(3HO)/P(3HB) (50:50) 5 μm fibers (91.48 ± 21.57 μm) and PCL 5 μm fibers (76.34 ± 14.03 μm). No significant difference was detected between P(3HO)/P(3HB) (50:50) 8 μm fibers and PCL 8 μm fibers.

### Primary Schwann Cell Metabolic Activity on Aligned Fibers with Varying Diameters

**Figure 7:**
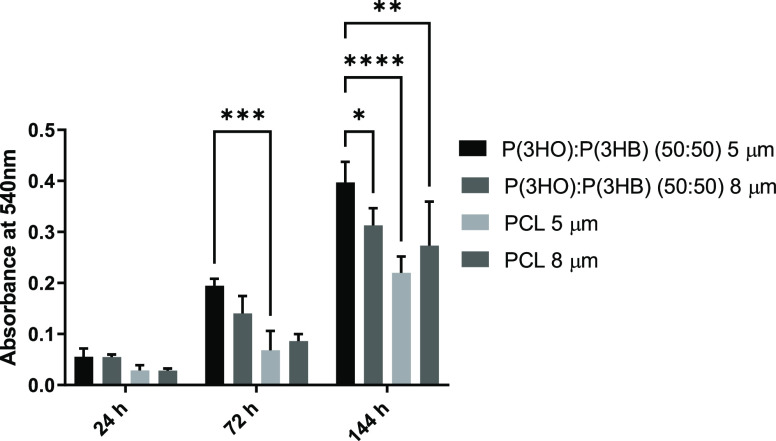
Metabolic viability assay (MTT) of rat primary Schwann cells on 5 and 8 μm P(3HO)/P(3HB) (50:50) and PCL fibers at 24, 72, and 144 h of culture (mean ± SD, *n* = 3 independent experiments; *p < 0.05, ***p < 0.001, and ****p < 0.001).

The metabolic activity of rat primary Schwann cells cultured on fibers was determined via an MTT assay (Figure 7). No significant difference was detected on samples after 24 h in culture. However, after 72 h, 5 μm PHA blend fibers supported significantly higher NG108-15 neuronal cell metabolic activity compared to PCL 5 μm fibers. After 144 h in the culture medium, 5 μm PHA blend fibers supported significantly higher rat primary Schwann cell metabolic activity compared to both the 5 and 8 μm PCL fibers and the 8 μm PHA blend fibers.

### Primary Schwann Cell Viability on Different Material Aligned Fibers with Varying Diameters

**Figure 8:**
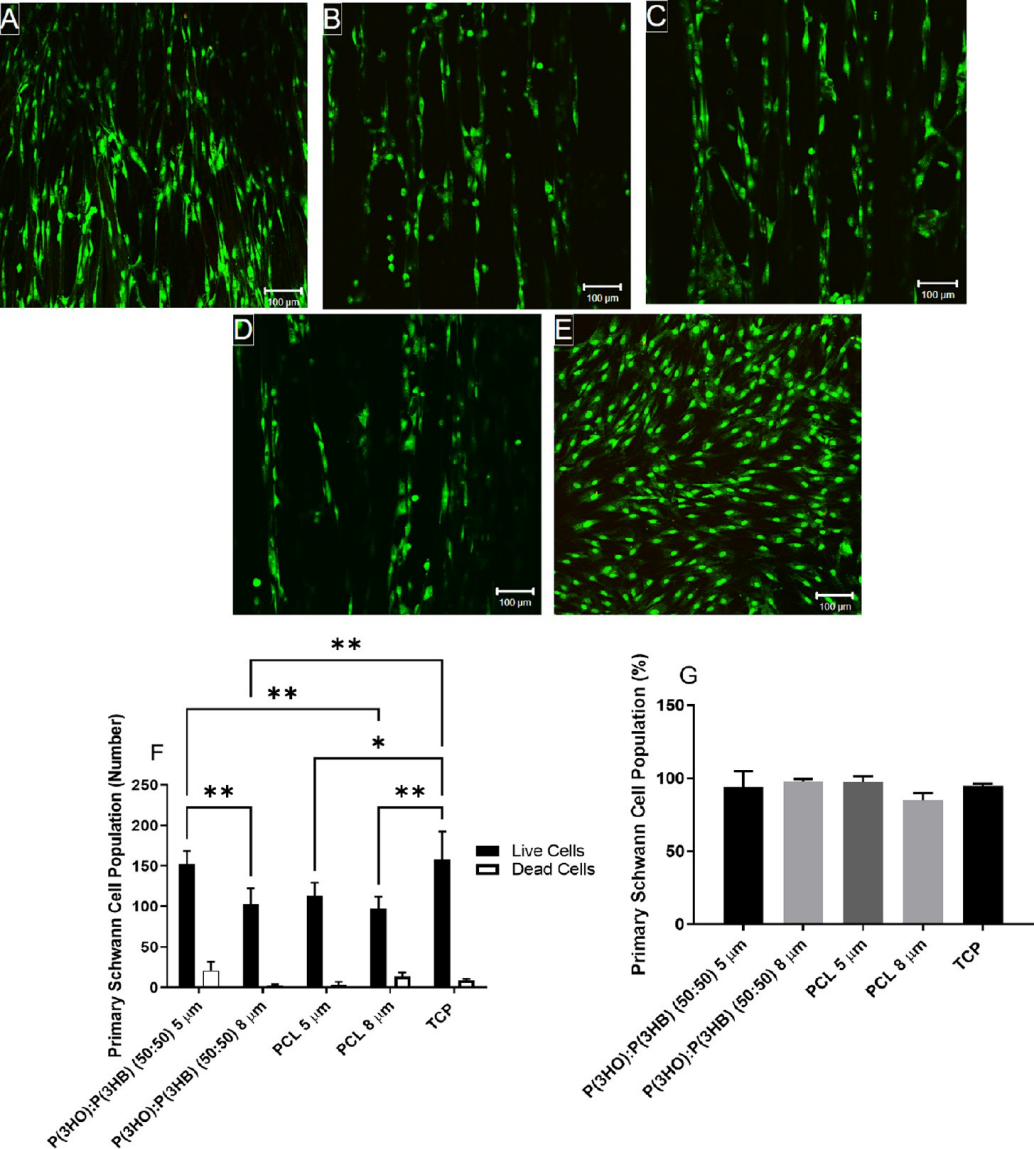
Confocal micrographs illustrating primary Schwann cell viability cultured on (A) P(3HO)/P(3HB) (50:50) 5 μm fibers, (B) P(3HO)/P(3HB) (50:50) 8 μm fibers, (C) PCL 5 μm fibers, (D) PCL 8 μm fibers, and (E) tissue culture plastic (scale bar = 100 μm). Cells labeled with SYTO 9 (green = live cells) and propidium iodide (red = dead cells). (F) Number of live cells versus dead cells per sample and (G) live cell analysis expressed as percentage (mean ± SD, *n* = 3 independent experiments; *p < 0.05 and **p < 0.01).

The electrospun aligned fibers supported primary Schwann cell attachment, and few dead cells were observed (Figure 8A–D). Schwann cells adhered and aligned themselves to polymer fibers in an elongated manner. The number of live Schwann cells attached on P(3HO)/P(3HB) (50:50) 5 μm fibers (152 ± 17 cells) was significantly higher than the number of live Schwann cells attached to the PCL 5 and 8 μm fibers and the P(3HO)/P(3HB) (50:50) 8 μm fibers (113 ± 16, 97 ± 25, and 102 ± 20 cells, respectively). No significant differences were detected between the material type and fiber diameter when expressed as cell viability percentage (Figure 8G). All conditions supported >85% cell viability.

### Primary Schwann Cell Culture on Different Material Aligned Fibers with Varying Diameters

**Figure 9:**
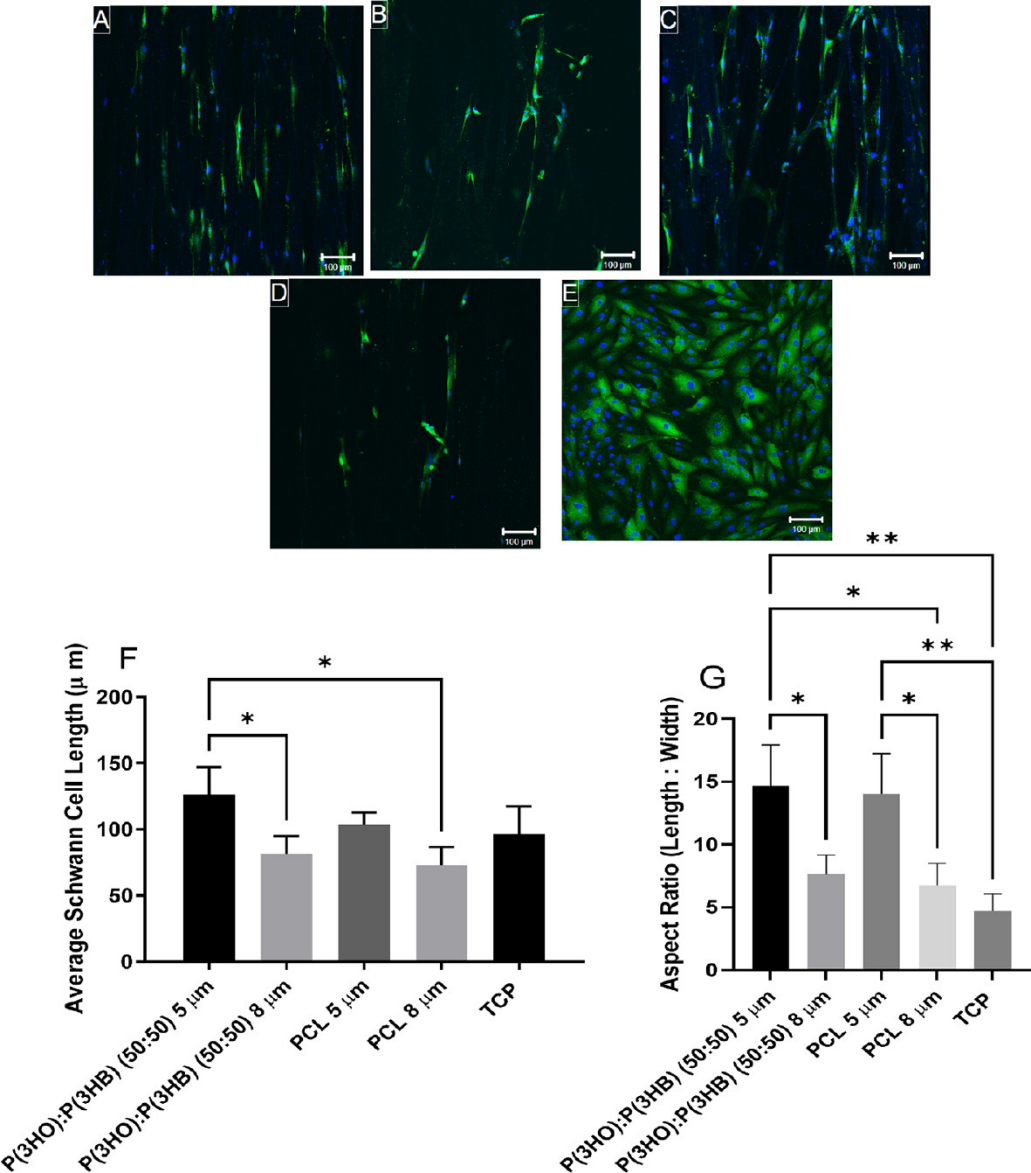
Confocal micrographs of primary Schwann cells immunolabeled for S100β and DAPI on (A) P(3HO)/P(3HB) (50:50) 5 μm fibers, (B) P(3HO)/P(3HB) (50:50) 8 μm fibers, (C) PCL 5 μm fibers, (D) PCL 8 μm fibers, and (E) tissue culture plastic (scale bar = 100 μm). Schwann cells were immunolabeled to confirm Schwann cell phenotype. (F) Average Schwann cell length. (G) Aspect ratio (length/width) to identify Schwann cell phenotype (mean ± SD, *n* = 3 independent experiments; *p < 0.05 and **p < 0.01).

Any changes in Schwann cell morphology when adhered to the polymer fibers were determined by calculating the average Schwann cell length and aspect ratio. Schwann cells stained positively for S100β, adhered to fibers, and had an elongated bipolar morphology, as seen in Figure 9A–D. Schwann cells expressed a more squamous morphology on TCP (Figure 9E). The average length of Schwann cells (Figure 9F) cultured on P(3HO)/P(3HB) (50:50) 5 μm fibers (126.09 ± 21.05 μm) was significantly higher than the average length of Schwann cells cultured on PCL and P(3HO)/P(3HB) (50:50) 8 μm fibers (72.78 ± 13.95 and 85.05 ± 4.87 μm, respectively). The average length of Schwann cells cultured on 5 μm PCL fibers and TCP surfaces was 103.25 ± 9.56 and 96.25 ± 21.05 μm. The aspect ratio (Figure 9G) of Schwann cells cultured on 5 μm fibers (PHA blend and PCL fibers) was significantly higher than the aspect ratio of cells cultured on their 8 μm counterparts. The aspect ratio of Schwann cells cultured on P(3HO)/P(3HB) (50:50) 5 μm fibers (14.65 ± 3.27 μm) was significantly higher than the aspect ratio of cells cultured on P(3HO)/P(3HB) (50:50) 8 μm fibers, PCL 8 μm fibers, and the TCP control (7.64 ± 1.52, 6.72 ± 1.78, and 4.71 ± 1.34 μm, respectively).

### Confirmation of Fiber Alignment and Volume Occupancy of Polymer Fibers Threaded into 3D Printed Nerve Guidance Conduits for *Ex Vivo* Analysis

**Figure 10:**
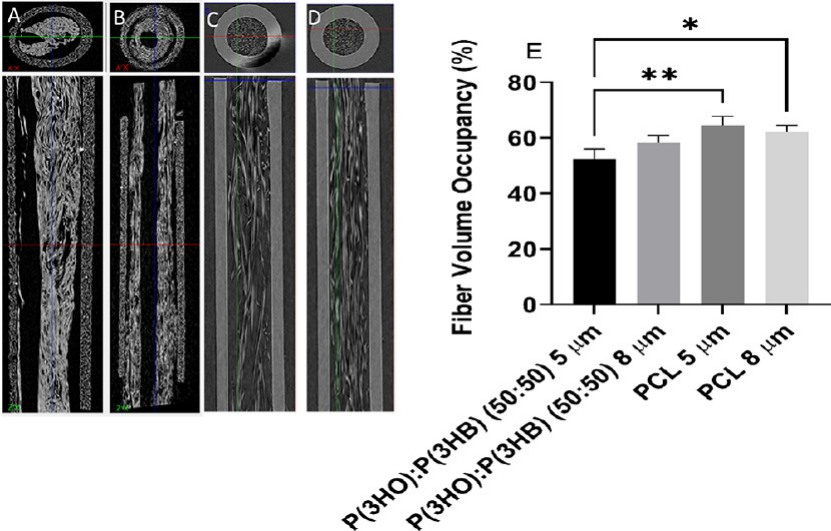
Microcomputed tomography (microCT) analysis of transversal and longitudinal sections of (A) P(3HO)/P(3HB) (50:50) 5 μm fibers, (B) P(3HO)/P(3HB) (50:50) 8 μm fibers, (C) PCL 5 μm fibers, and (D) PCL 8 μm fibers threaded into 3D nerve guide conduits. Tubular structures are 1.1 mm in diameter and 5 mm in length and have a wall thickness of 250 μm. (E) The percentage volume occupancy of the fibers in the NGC lumen was calculated by calculating the density of fibers and density of the empty tube and expressed as a percentage (mean ± SD, *n* = 3 independent experiments; *p < 0.05 and **p < 0.01).

### Mechanical Testing

The mechanical data of electrospun fiber bundles of different diameters are shown in Table 3. With regard to the ultimate tensile strength (UTS), 5 μm PCL fiber bundles had a significantly higher UTS than their 5 μm PHA blend fiber counterparts (6.09 ± 0.16 compared to 2.69 ± 0.27 MPa, respectively). No significant difference was found between the ultimate tensile strength of 8 μm PCL and PHA blend fibers. However, with regard to Young’s modulus (YM), PCL fiber bundles had significantly higher Young’s moduli compared to their PHA blend fiber counterparts. The 5 μm PCL fibers had a YM of 9.18 ± 0.18 MPa, whereas the YM of 5 μm PHA fibers was 7.45 ± 0.36 MPa. The YM of 8 μm PCL fiber bundles was 8.87 ± 0.25 MPa, whereas it was 6.75 ± 0.05 MPa for 8 μm PHA fiber bundles. Of note, all YM values for all fibers and materials were significantly higher than that reported for native rat sciatic nerve of 0.57–0.58 MPa.^27^

**Table 3:** Mechanical Analysis of the Electrospun Fiber Material Bundles

fibers and size	σ, MPa	*E*, MPa
P(3HO)/P(3HB) (50:50) 5 μm	2.69 ± 0.27	7.45 ± 0.36
P(3HO)/P(3HB) (50:50) 8 μm	4.40 ± 0.83	6.75 ± 0.05
PCL 5 μm	6.09 ± 0.16	9.18 ± 0.18
PCL 8 μm	4.53 ± 0.49	8.87 ± 0.25
rat sciatic nerve	1.4–2.7	0.57–0.58

### Primary Schwann Cell Migration and Neurite Outgrowth from Dorsal Root Ganglia on Aligned Fibers with Varying Diameters

**Figure 11:**
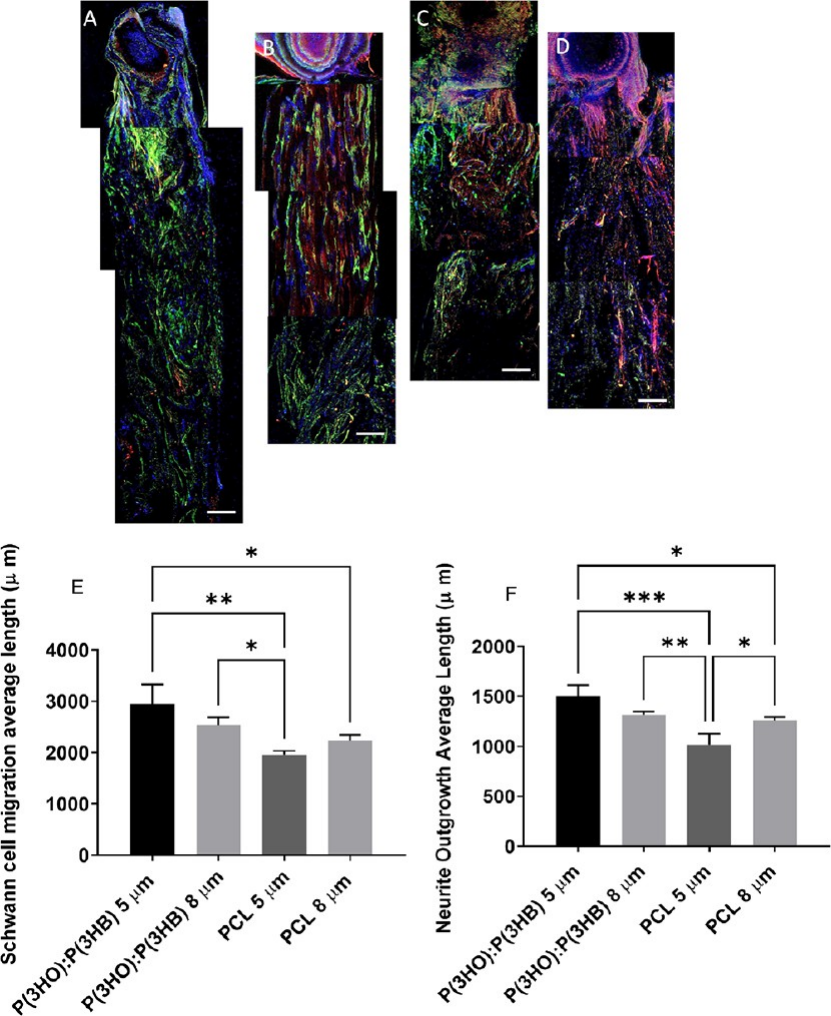
Confocal micrographs DRG explants on fibers immunolabeled for βIII tubulin, S100β, and DAPI on (A) P(3HO)/P(3HB) (50:50) 5 μm fibers, (B) P(3HO)/P(3HB) (50:50) 8 μm fibers, (C) PCL 5 μm fibers, and (D) PCL 8 μm fibers (scale bar = 200 μm). (E) Schwann cell migration length and (F) average neurite outgrowth length were determined from confocal micrographs (mean ± SD, *n* = 3 independent experiments; *p < 0.05, **p < 0.01, and ***p < 0.001).

The effect of fiber diameter and material type on neurite outgrowth length and Schwann cell migration length from DRG explants was determined using confocal micrographs. Schwann cells could be visualized as being the furthest away from the DRG body on both diameters of PHA blend fibers (Figure 11A,B) and the TCP control (Figure 11E) compared to PCL fibers (Figure 11C,D). Importantly, neurite outgrowth (red) and Schwann cells (green) were co-located, indicating neurite–glial cell contact. Average Schwann cell migration distance (Figure 11F) was significantly higher on P(3HO)/P(3HB) (50:50) 5 μm fibers (2943.58 ± 386.09 μm) compared to 5 and 8 μm PCL fibers (1959.99 ± 76.54 and 2237.75 ± 108.62 μm, respectively). Schwann cell migration distance was significantly lower on PCL 5 μm fibers compared to P(3HO)/P(3HB) (50:50) 8 μm fibers and TCP control (2723.20 ± 86.05 μm). Average neurite outgrowth length (Figure 11G) from DRG explants was significantly further on P(3HO)/P(3HB) (50:50) 5 μm fibers (1502.69 ± 110.63 μm) and TCP control (1416.95 ± 31.48 μm) compared to PCL 5 μm fibers (1016.95 ± 110.63 μm). Neurite outgrowth length on P(3HO)/P(3HB) (50:50) 8 μm fibers and 8 μm PCL fibers was 1316.95 ± 31.48 and 1262.69 ± 31.48 μm, respectively.

## Discussion

This study investigated the effect of fiber diameter as well as material chemistry on neuronal and Schwann cell viability, neuronal cell differentiation, and Schwann cell phenotype using 2D *in vitro* and 3D *ex vivo* cell culture. A previous work has highlighted the potential of PHA blends in peripheral nerve repair and demonstrated favorable responses on NG108-15 neuronal cell biocompatibility and differentiation compared to PCL.^26^

Prior to electrospinning, spin-coated films of both material types were manufactured to assess surface characteristics, such as hydrophilicity, topography and surface roughness. PHA films exhibited a hydrophilic nature, having a water contact angle just lower than 90°, whereas PCL films had a water contact angle above 90°, indicating that they were more hydrophobic in nature. Our previous study noted that water contact angles for P(3HO) and P(3HB) were 103.7 ± 0.9 and 69.7 ± 1.6°, respectively, in which increasing the amount of P(3HB) in the blend significantly decreased water contact angle.^26^ P(3HB) is known to be more hydrophilic in nature compared to P(3HO) due to having less carbon atoms (four) in the monomer compared to the P(3HO) monomer (eight).^28^ The water contact angle recorded for P(3HB)/P(3HO), 84.3 ± 1.6°, is comparable with that of other findings in the literature.^19,25^

AFM analysis confirmed that PHA blend films were significantly rougher than the PCL films, indicated by Rq and Ra values, and PHA films had increased adhesion, exhibiting areas of both low and high adhesion, due to both polymers being immiscible. This was also noted in the study by Lizarraga-Valderrama *et al.* where blends of P(3HB) and P(3HO) were found to be immiscible in both the amorphous and crystalline phases, when blend films were analyzed using X-ray diffraction analysis, compared to neat films of P(3HO) and P(3HB).^26^ Basnett *et al.* also investigated the surface roughness of P(3HO)/P(3HB) (50:50) blend compared to neat P(3HO), suggesting that the increased roughness of the 50:50 blend was due to the P(3HB) “generating protrusions and pores” during the casting process.^20^ As PCL is a single polymer, the result of a smoother surface topography is expected.

P(3HO)/P(3HB) (50:50) blends also significantly promoted protein adsorption of proteins in the fetal calf serum compared to PCL films. Similar observations were reported by Basnett *et al.* who reported increased protein adsorption in the PHA blends compared to neat P(3HO) and P(3HB) due to the increased surface roughness due to immiscible polymers in the blend, as well as an increase in hydrophilicity when incorporating larger quantities of P(3HB) into the blend.^20^ It is well documented that rougher surface topographies increase protein adsorption and hydrophilic surfaces retain native protein confirmation, both factors favoring an increase in cellular adhesion and in turn cell differentiation.^37,38^ Both PCL and P(3HO) are hydrophobic polymers due to the presence of long aliphatic chains in their monomer backbone.^20,39^ The addition of P(3HB) to the blend increases its hydrophilicity, as seen in previously published studies.^20,26^

Aligned fibers of P(3HO)/P(3HB) (50:50) were fabricated by electrospinning. Diameters of 5 and 8 μm were chosen for this study, as previous work using PCL fibers concluded that these diameters significantly supported NG108-15 neuronal cell viability and differentiation, Schwann cell viability, longer maximum neurite outgrowth, and Schwann cell migration length from DRG explants.^16^ PCL fibers (5 and 8 μm) were also manufactured as a positive control to compare neuronal/glial cell responses to material type. Figure 4E confirms the fabrication of P(3HO)/P(3HB) (50:50) fibers with two significantly different diameters (4.97 ± 0.38 and 7.86 ± 0.49 μm). All electrospinning conditions resulted in highly aligned fibers running parallel to each other, and studies observed a significantly higher fiber density (fibers/μm) for 8 μm compared to 5 μm fibers.

PHA blend fibers were found to better support NG108-15 neuronal cell proliferation over 6 days compared to PCL 5 fibers (Figure 5). PHA blend fibers were also found to better support a higher number of live cells compared to the 5 μm PCL fibers. This indicated that the PHA blend was superior in supporting cell proliferation and viability and suggested initial cell adhesion, which would have to be investigated further using AFM, for example. These findings can be attributed to the blend having higher roughness values and lower hydrophobicity compared to PCL when in film form (Figures 1 and 2). These results are in line with our previous findings.^26^ No significant differences were detected between fiber diameters per material type, an observation also observed by Daud *et al*., who did not detect significant differences in the number of live cells adhered to 5 and 8 μm PCL fibers.^16^ Of note, TCP had the lowest cell viability of 90.38 ± 4.82% likely due to the increased cell numbers. However, all samples were deemed biocompatible.

NG108-15 neuronal cell average neurite length was affected by fiber diameter. Regardless of the material type, 8 μm fibers significantly promoted longer average neurite lengths compared to their 5 μm counterparts, findings that are consistent with previous studies concluding that larger fiber diameters support longer average neurite lengths.^16,40^ The longer neurite growth from NG108-15 neuronal cells cultured on PHA fibers compared to PCL fibers could be due to a combination of differences in surface roughness, chemical composition, and mechanical properties of the materials. Differences in surface topography have been shown to control neuronal cell differentiation.^41^ Lizarraga-Valderrama *et al.* reported that rougher surfaces promoted NG108-15 neuronal cell differentiation, and Zhang *et al.* reported significantly longer neurites outgrown from PC12 cells on rougher PLLA fibers containing graphene oxide compared to smoother PLLA fibers.^26,42^ Material stiffness has also been shown to control neuronal cell differentiation.^43^ Palchesko *et al.* reported longer neurites from PC12 cells cultured on stiff polydimethylsiloxane (PDMS) substrates (1.72 MPa) compared to soft substrates (5 kPa).^44^ Rosso *et al.* reported significantly longer neurite outgrowth from dorsal root ganglia explants on stiff substrates (20 kPa) compared to softer substrates (1 and 10 kPa).^45^ Both materials, regardless of fiber diameter, have significantly higher ultimate tensile strength and Young’s moduli compared to rat sciatic nerve and are much stiffer.^26^ Fiber spacing could also have an effect on the neurite outgrowth of NG108-15 neuronal cells. Both PHA blend and PCL 8 μm fibers had a significantly higher fiber frequency compared to the 5 μm fibers, which could be responsible for the increased average neurite lengths measured. However, these measurements were recorded in dry environments, whereas it is well known that fiber alignment, spacing, and density can change in a wet environment.

Material chemistry and fiber diameter have also been shown to influence primary Schwann cell response. P(3HO)/P(3HB) (50:50) 5 μm fibers significantly supported the adhesion of higher numbers of live Schwann cells compared to P(3HO)/P(3HB) (50:50) 8 μm fibers and both diameters of PCL fibers. This is in line with previous findings that conclude that fiber diameters between 1 and 5 μm using PCL fibers support Schwann cell adhesion, proliferation, and migration to a greater extent than other fiber diameters .^13,16^ This is the first study of its kind to investigate PHA blend fibers and different diameters using primary Schwann cells. A previous work has investigated Schwann cell response using immortal Schwann cells; however, these are not always representative of the *in vivo* response.^16^ Surface roughness, stiffness, and hydrophilicity have all been shown to increase Schwann cell adhesion and proliferation, which are also in line with our findings.^45,46^

Aligned fiber scaffolds have previously been shown to affect Schwann cell phenotype, promoting a more elongated “mature” phenotype compared to random scaffolds.^9^ We investigated whether material type and fiber diameter would also affect Schwann cell phenotype. PHA blend fibers significantly promoted longer elongated Schwann cells compared to PCL fibers, and 5 μm fibers promoted longer and thinner Schwann cells compared to 8 μm fibers. This is in line with previous studies that have shown that smaller fiber diameters promote an elongated bipolar Schwann cell phenotype, an indication of Schwann cell maturity.^9,16^ Schwann cells with a long, elongated phenotype are observed in the early stages of myelination *in vivo*.^47^ By promoting a mature myelinating phenotype, electrospun fibers could therefore increase the nerve regeneration rate by the remyelination of regenerated nerve tissue.^10^ The effect of material chemistry and fiber diameter was evaluated using a novel 3D *ex vivo* fiber testing method developed by Behbehani *et al.* using DRG explants as a primary cell peripheral nerve injury model.^7^

The use of DRG explants to assess the suitability of materials and scaffold designs for peripheral nerve repair is a relatively common approach *in vitro* prior to *in vivo* investigations and in line with the 3R principle.^49^ By removing the nerve roots from the body, mimicking an injury, ganglia regenerate and repair *in vitro*, and regeneration is comparable to the *in vivo* response.^31^ Previous studies have conducted DRG investigations on electrospun fibers in 2D environments, whereas our model replicates a 3D environment, more closely mimicking the *in vivo* environment investigating an NGC with an intraluminal scaffold, to bridge the gap between *in vitro* and *in vivo* testing.^7^ Fibers were threaded into 3D printed PEG tubes and evaluated for continuous alignment using microCT before *ex vivo* cell culture.

Because of the elastomeric properties of P(3HO), clumping of PHA fibers was observed when threading the fibers into 3D printed PEG NGCs, resulting in a hole in the middle of the NGC lumen when using the 8 μm PHA blend fibers; therefore, uniformity of threading did not occur, which is a hindrance of the 3D *ex vivo* model. However, the softer, “stickier” properties of the PHA fibers may be advantageous for peripheral nerve repair when threaded into an NGC lumen, as they have a lower lumen occupancy percentage, preventing blocking of the axon outgrowth.

Overall, P(3HO)/P(3HB) (50:50) 5 μm fibers supported significantly higher Schwann cell migration distances compared to 5 or 8 μm PCL fibers, most likely as a result of an increase in viable primary Schwann cells (Figure 9) and elongated bipolar morphology (Figure 10). P(3HO)/P(3HB) (50:50) 5 μm fibers also supported significantly longer neurite outgrowth length from DRG explants compared to PCL 5 μm fibers. As the PHA blend supported increased NG108-15 neuronal cell attachment and increased average neurite outgrowth length, this finding was expected. However, when using the NG108-15 neuronal cell line, 8 μm PHA fibers supported longer neurite outgrowth length compared to the 5 μm fibers. This finding is likely due to the difference in using 2D cell culture with the NG108-15 neuronal cells compared to using a 3D model using the DRG explants. It also highlights the importance of using *ex vivo* explants and primary cells—compared to immortal cell lines—that more closely resemble the *in vivo* response prior to *in vivo* investigations. It is also likely that fiber spacing plays a role in cell responses *ex vivo* in which, in a wet environment, fibers clump together.

This study is the first of its kind to investigate fibers manufactured using blends of PHAs for nerve tissue engineering using *ex vivo* DRG explants and in a 3D testing model. The 5 μm PHA blend fibers supported significantly longer neurite outgrowth compared to 5 μm PCL fibers likely because of differences in material chemistry, but this finding could also be due to differences in mechanical properties of the materials and fiber volume occupancy (Figure 8E). Although it has been well reported that the addition of guidance scaffolds to hollow NGCs increases nerve regeneration rate and distance, “overfilling” the lumen, as well as using stiff material guidance scaffolds, could physically hinder nerve regeneration by not providing sufficient space for regenerating axons.^6^ Our finding initially suggests that using a lower volume occupancy percent of fibers in the NGC lumen does not hinder Schwann cell migration or neurite outgrowth from DRG explants, allowing enough free space. As noted previously, clumping of the PHA fibers when threaded could have allowed more free lumen space, aiding the results obtained. Our finding may also suggest that using a softer material, such as blends of PHAs, compared to PCL supports Schwann cell migration and axon outgrowth. However, further work is required to establish an optimal available lumen volume, for both *in vitro* and *in vivo* investigations, as well as reproducible ways to control fiber properties in a wet environment.

## Conclusions

In summary, we report the potential value of aligned PHA fiber blend fibers for nerve repair devices. P(3HO)/P(3HB) (50:50) blend fibers, 5 and 8 μm in diameter, supported increased neuronal and Schwann cell attachment compared to PCL fibers as a result of differences in surface wettability, roughness, and mechanical properties. Smaller fiber diameters (5 μm) supported increased Schwann cell attachment and supported a bipolar elongated Schwann cell morphology, characteristic of mature myelinating Schwann cells *in vivo*. Using a novel 3D *ex vivo* testing method, P(3HO)/P(3HB) (50:50) 5 μm fibers supported longer neurite outgrowth and Schwann cell migration lengths from DRG explants. Overall, this study highlights the positive properties of polyhydroxyalkanoates as compared to PCL for applications in peripheral nerve repair.

## ABBREVIATIONS

AFM: atomic force microscopy
DMEM: Dulbecco’s modified Eagle’s medium
DRG: dorsal root ganglia
MicroCT: microcomputed tomography
PNI: peripheral nerve injury
PCL: polycaprolactone
PEG: polyethylene glycol
PHAs: polyhydroxyalkanoates
P(3HB): poly-3hydroxybutyrate
P(3HO): poly-3hydroxyoctanoate
NGCs: nerve guide conduits
TCP: tissue culture plastic
WCA: water contact angle

## References

[ref1] Bell J. H. A.; Haycock J. W. Next generation nerve guides: materials, fabrication, growth factors, and cell delivery. Tissue Eng., Part B 2012, 18, 116–128. 10.1089/ten.TEB.2011.0498. 22010760

[ref2] Nectow A. R.; Marra K. G.; Kaplan D. L. Biomaterials for the development of peripheral nerve guidance conduits. Tissue Eng., Part B 2012, 18, 40–50. 10.1089/ten.TEB.2011.0240. PMC3262974 21812591

[ref3] Singh D.; Harding A. J.; Albadawi E.; Boissonade F. M.; Haycock J. W.; Claeyssens F. Additive manufactured biodegradable poly(glycerol sebacate methacrylate) nerve guidance conduits. Acta Biomater. 2018, 78, 48–63. 10.1016/j.actbio.2018.07.055. 30075322

[ref4] Daly W.; Yao L.; Zeugolis D.; Windebank A.; Pandit A. A biomaterials approach to peripheral nerve regeneration: bridging the peripheral nerve gap and enhancing functional recovery. J. R. Soc., Interface 2012, 9, 202–221. 10.1098/rsif.2011.0438. 22090283 PMC3243399

[ref5] Spivey E. C.; Khaing Z. Z.; Shear J. B.; Schmidt C. E. The fundamental role of subcellular topography in peripheral nerve repair therapies. Biomaterials 2012, 33, 4264–4276. 10.1016/j.biomaterials.2012.02.043. 22425024

[ref6] Carvalho C. R.; Oliveira J. M.; Reis R. L. Modern Trends for Peripheral Nerve Repair and Regeneration: Beyond the Hollow Nerve Guidance Conduit. Front. Bioeng. Biotechnol. 2019, 7, 337 10.3389/fbioe.2019.00337. 31824934 PMC6882937

[ref7] Behbehani M.; Glen A.; Taylor C. S.; Schuhmacher A.; Claeyssens F.; Haycock J. W. Pre-clinical evaluation of advanced nerve guide conduits using a novel 3D in vitro testing model. Int. J. Bioprint. 2018, 4, 1–12. 10.18063/ijb.v4i1.123. PMC7582002 33102907

[ref8] Frost H. K.; Andersson T.; Johansson S.; Englund-Johansson U.; Ekström P.; Dahlin L. B.; Johansson F. Electrospun nerve guide conduits have the potential to bridge peripheral nerve injuries in vivo. Sci. Rep. 2018, 8, 16716 10.1038/s41598-018-34699-8. 30425260 PMC6233209

[ref9] Chew S. Y.; Mi R.; Hoke A.; Leong K. W. The effect of the alignment of electrospun fibrous scaffolds on Schwann cell maturation. Biomaterials 2008, 29, 653–661. 10.1016/j.biomaterials.2007.10.025. 17983651 PMC2713097

[ref10] Jessen K. R.; Mirsky R.; Lloyd A. C. Schwann Cells: Development and Role in Nerve Repair. Cold Spring Harbor Perspect. Biol. 2015, 7, a020487 10.1101/cshperspect.a020487. PMC4484967 25957303

[ref11] Corey J. M.; Lin D. Y.; Mycek K. B.; Chen Q.; Samuel S.; Feldman E. L.; Martin D. C. Aligned electrospun nanofibers specify the direction of dorsal root ganglia neurite growth. J. Biomed. Mater. Res., Part A 2007, 83A, 636–645. 10.1002/jbm.a.31285. 17508416

[ref12] Kim Y. T.; Haftel V. K.; Kumar S.; Bellamkonda R. V. The role of aligned polymer fiber-based constructs in the bridging of long peripheral nerve gaps. Biomaterials 2008, 29, 3117–3127. 10.1016/j.biomaterials.2008.03.042. 18448163 PMC2483242

[ref13] Gnavi S.; Fornasari B. E.; Tonda-Turo C.; Ciardelli G.; Zanetti M.; Geuna S.; Perroteau I. The influence of electrospun fibre size on Schwann cell behaviour and axonal outgrowth. Mater. Sci. Eng., C 2015, 48, 620–631. 10.1016/j.msec.2014.12.055. 25579965

[ref14] Yang F.; Murugan R.; Wang S.; Ramakrishna S. Electrospinning of nano/micro scale poly(L-lactic acid) aligned fibers and their potential in neural tissue engineering. Biomaterials 2005, 26, 2603–2610. 10.1016/j.biomaterials.2004.06.051. 15585263

[ref15] Wang H. B.; Mullins M. E.; Cregg J. M.; McCarthy C. W.; Gilbert R. J. Varying the diameter of aligned electrospun fibers alters neurite outgrowth and Schwann cell migration. Acta Biomater. 2010, 6, 2970–2978. 10.1016/j.actbio.2010.02.020. 20167292

[ref16] Daud M. F. B.; Pawar K. C.; Claeyssens F.; Ryan A. J.; Haycock J. W. An aligned 3D neuronal-glial co-culture model for peripheral nerve studies. Biomaterials 2012, 33, 5901–5913. 10.1016/j.biomaterials.2012.05.008. 22656449

[ref17] Lizarraga-Valderrama L. R.; Taylor C. S.; Claeyssens F.; Haycock J. W.; Knowles J. C.; Roy I. Unidirectional neuronal cell growth and differentiation on aligned polyhydroxyalkanoate blend microfibres with varying diameters. J. Tissue Eng. Regener. Med. 2019, 13, 1581–1594. 10.1002/term.2911. PMC6790610 31185133

[ref18] Kehoe S.; Zhang X. F.; Boyd D. FDA approved guidance conduits and wraps for peripheral nerve injury: a review of materials and efficacy. Injury. 2012, 43, 553–572. 10.1016/j.injury.2010.12.030. 21269624

[ref19] Basnett P.; Lukasiewicz B.; Marcello E.; Gura H. K.; Knowles J. C.; Roy I. Production of a novel medium chain length poly(3-hydroxyalkanoate) using unprocessed biodiesel waste and its evaluation as a tissue engineering scaffold. Microb. Biotechnol. 2017, 10, 1384–1399. 10.1111/1751-7915.12782. 28905518 PMC5658593

[ref20] Basnett P.; Ching K. Y.; Stolz M.; Knowles J. C.; Boccaccini A. R.; Smith C.; Locke I. C.; Keshavarz T.; Roy I. Novel Poly(3-hydroxyoctanoate)/Poly(3-hydroxybutyrate) blends for medical applications. React. Funct. Polym. 2013, 73, 1340–1348. 10.1016/j.reactfunctpolym.2013.03.019.

[ref21] Prabhakaran M. P.; Vatankhah E.; Ramakrishna S. Electrospun aligned PHBV/collagen nanofibers as substrates for nerve tissue engineering. Biotechnol. Bioeng. 2013, 110, 2775–2784. 10.1002/bit.24937. 23613155

[ref22] Hazer D. B.; Bal E.; Nurlu G.; Benli K.; Balci S.; Öztürk F.; Hazer B. In vivo application of poly-3-hydroxyoctanoate as peripheral nerve graft. J. Zhejiang Univ., Sci., B 2013, 14, 993–1003. 10.1631/jzus.B1300016. 24190445 PMC3829648

[ref23] Young R. C.; Wiberg M.; Terenghi G. Poly-3-hydroxybutyrate (PHB): a resorbable conduit for long-gap repair in peripheral nerv. J. Plast. Surg. 2002, 55, 235–240. 10.1054/bjps.2002.3798. 12041978

[ref24] Mosahebi A.; Wiberg M.; Terenghi G. Addition of fibronectin to alginate matrix improves peripheral nerve regeneration in tissue-engineered conduits. Tissue Eng. 2003, 9, 209–218. 10.1089/107632703764664684. 12740084

[ref25] Ching K. Y.; Andriotis O. G.; Li S.; Basnett P.; Su B.; Roy I.; Tare R. S.; Sengers B. G.; Stolz M. Nanofibrous poly(3-hydroxybutyrate)/poly(3-hydroxyoctanoate) scaffolds provide a functional microenvironment for cartilage repair. J. Biomater. Appl. 2016, 31, 77–91. 10.1177/0885328216639749. 27013217

[ref26] Lizarraga-Valderrama L. R.; Nigmatullin R.; Taylor C.; Haycock J. W.; Claeyssens F.; Knowles J. C.; Roy I. Nerve tissue engineering using blends of poly(3-hydroxyalkanoates) for peripheral nerve regeneration. Eng. Life Sci. 2015, 15, 612–621. 10.1002/elsc.201400151.

[ref27] Borschel G. H.; Kia K. F.; Kuzon W. M. Jr.; Dennis R. G. Mechanical properties of acellular peripheral nerve. J. Surg. Res. 2003, 114, 133–139. 10.1016/S0022-4804(03)00255-5. 14559438

[ref28] Valappil S. P.; Peiris D.; Langley G. J.; Herniman J. M.; Boccaccini A. R.; Bucke C.; Roy I. Polyhydroxyalkanoate (PHA) biosynthesis from structurally unrelated carbon sources by a newly characterized Bacillus spp. J. Biotechnol. 2007, 127, 475–487. 10.1016/j.jbiotec.2006.07.015. 16956686

[ref29] Rai R.; Yunos D. M.; Boccaccini A. R.; Knowles J. C.; Barker I. A.; Howdle S. M.; Tredwell G. D.; Keshavarz T.; Roy I. Poly-3-hydroxyoctanoate P(3HO), a medium chain length polyhydroxyalkanoate homopolymer from Pseudomonas mendocina. Biomacromolecules 2011, 12, 2126–2136. 10.1021/bm2001999. 21561067

[ref30] Kaewkhaw R.; Scutt A. M.; Haycock J. W. Integrated culture and purification of rat Schwann cells from freshly isolated adult tissue. Nat. Protoc. 2012, 7, 1996–2004. 10.1038/nprot.2012.118. 23060244

[ref31] Pateman C. J.; Harding A. J.; Glen A.; Taylor C. S.; Christmas C. R.; Robinson P. P.; Rimmer S.; Biossonade F. M.; Claeyssens F.; Haycock J. W. Nerve guides manufactured from photocurable polymers to aid peripheral nerve repair. Biomaterials 2015, 49, 77–89. 10.1016/j.biomaterials.2015.01.055. 25725557

[ref32] Usaj M.; Torkar D.; Kanduser M.; Miklavcic D. Cell counting tool parameters optimization approach for electroporation efficiency determination of attached cells in phase contrast images. J. Microsc. 2011, 241, 303–314. 10.1111/j.1365-2818.2010.03441.x. 21118234

[ref33] Diez-Ahedo R.; Mendibil X.; Márquez-Posadas C. M.; Quintana I.; González F.; Rodríguez J. F.; Zilic L.; Sherborne C.; Glen A.; Taylor C. S.; Claeyssens F.; Haycock J. W.; Schaafsma W.; González E.; Castro B.; Merino S. UV-Casting on Methacrylated PCL for the Production of a Peripheral Nerve Implant Containing an Array of Porous Aligned Microchannels. Polymer 2020, 12, 971 10.3390/polym12040971. PMC7240584 32331241

[ref34] Schneider C. A.; Rasband W. S.; Eliceiri K. W. NIH Image to ImageJ: 25 years of image analysis. Nat. Methods 2012, 9, 671 10.1038/nmeth.2089. 22930834 PMC5554542

[ref35] Popko J.; Fernandes A.; Brites D.; Lanier L. M. Automated Analysis of NeuronJ Tracing Data. Cytometry, Part A 2009, 75A, 371–376. 10.1002/cyto.a.20660. PMC2661008 18937344

[ref36] Kaewkhaw R.; Scutt A. M.; Haycock J. W. Anatomical site influences the differentiation of adipose-derived stem cells for Schwann-cell phenotype and function. Glia. 2011, 59, 734–749. 10.1002/glia.21145. 21351157

[ref37] Anselme K.; Ploux L.; Ponche A. Cell/Material Interfaces: Influence of Surface Chemistry and Surface Topography on Cell Adhesion. J. Adhes. Sci. Technol. 2010, 24, 831–852. 10.1163/016942409X12598231568186.

[ref38] Colley H. E.; Mishra G.; Scutt A. M.; McArthur S. L. Plasma Polymer Coatings to Support Mesenchymal Stem Cell Adhesion, Growth and Differentiation on Variable Stiffness Silicone Elastomers. Plasma Processes Polym. 2009, 6, 831–839. 10.1002/ppap.200900040.

[ref39] Ulery B. D.; Nair L. S.; Laurencin C. T. Biomedical Applications of Biodegradable Polymers. J. Polym. Sci., Part B: Polym. Phys. 2011, 49, 832–864. 10.1002/polb.22259. PMC3136871 21769165

[ref40] Yao L.; O’Brien N.; Windebank A.; Pandit A. Orienting neurite growth in electrospun fibrous neural conduits. J. Biomed. Mater. Res., Part B 2009, 90B, 483–491. 10.1002/jbm.b.31308. 19130615

[ref41] Roach P.; Parker T.; Gadegaard N.; Alexander M. R. Surface strategies for control of neuronal cell adhesion: A review. Surf. Sci. Rep. 2010, 65, 145–173. 10.1016/j.surfrep.2010.07.001.

[ref42] Zhang K.; Zheng H.; Liang S.; Gao C. Aligned PLLA nanofibrous scaffolds coated with graphene oxide for promoting neural cell growth. Acta Biomater. 2016, 37, 131–142. 10.1016/j.actbio.2016.04.008. 27063493

[ref43] Kayal C.; Moeendarbary E.; Shipley R. J.; Phillips J. B. Mechanical Response of Neural Cells to Physiologically Relevant Stiffness Gradients. Adv. Healthcare Mater. 2020, 9, 1901036 10.1002/adhm.201901036. PMC8407326 31793251

[ref44] Palchesko R. N.; Zhang L.; Sun Y.; Feinberg A. W. Development of polydimethylsiloxane substrates with tunable elastic modulus to study cell mechanobiology in muscle and nerve. PLoS One 2012, 7, e51499 10.1371/journal.pone.0051499. 23240031 PMC3519875

[ref45] Rosso G.; Liashkovich I.; Young P.; Röhr D.; Shahin V. Schwann cells and neurite outgrowth from embryonic dorsal root ganglions are highly mechanosensitive. Nanomedicine 2017, 13, 493–501. 10.1016/j.nano.2016.06.011. 27389149

[ref46] Fonner J. M.; Forciniti L.; Nguyen H.; Byrne J. D.; Kou Y.-F.; Syeda-Nawaz J.; Schmidt C. E. Biocompatibility implications of polypyrrole synthesis techniques. Biomed. Mater. 2008, 3, 034124 10.1088/1748-6041/3/3/034124. 18765899 PMC2562301

[ref47] Deumens R.; Bozkurt A.; Meek M. F.; Marcus M. A. E.; Joosten E. A. J.; Weis J.; Brook G. A. Repairing injured peripheral nerves: Bridging the gap. Prog. Neurobiol. 2010, 92, 245–276. 10.1016/j.pneurobio.2010.10.002. 20950667

[ref49] Tannenbaum J.; Bennett B. T. Russell and Burch’s 3Rs Then and Now: The Need for Clarity in Definition and Purpose. J. Am. Assoc. Lab. Anim. Sci. 2015, 54, 120–132. 25836957 PMC4382615

